# Hepatitis-B-Virus-Infektionen und impfinduzierte Immunität: die Rolle von soziodemografischen Determinanten: Ergebnisse der „Studie zur Gesundheit Erwachsener in Deutschland“ (DEGS1, 2008–2011)

**DOI:** 10.1007/s00103-021-03473-z

**Published:** 2021-12-27

**Authors:** Annika Brodzinski, Angela Neumeyer-Gromen, Sandra Dudareva, Ruth Zimmermann, Ute Latza, Viviane Bremer, Christina Poethko-Müller

**Affiliations:** 1 Institut für Hygiene und Umweltmedizin, Charité – Universitätsmedizin Berlin, Berlin, Deutschland; 2 Deutsche Krankenhausgesellschaft e. V. (DKG), Berlin, Deutschland; 3 Abteilung für Infektionsepidemiologie, Robert Koch-Institut, Berlin, Deutschland; 4 Fachbereich Arbeit und Gesundheit, Bundesanstalt für Arbeitsschutz und Arbeitsmedizin BAuA, Berlin, Deutschland; 5 Abteilung für Epidemiologie und Gesundheitsmonitoring, FG 25 Körperliche Gesundheit, Robert Koch-Institut, General-Pape-Straße 62–66, 12101 Berlin, Deutschland

**Keywords:** Hepatitis B, Epidemiologie, Impfung, Prävalenz, Deutschland, Hepatitis B, Epidemiology, Vaccination, Prevalence, Germany

## Abstract

**Hintergrund und Ziel:**

Trotz niedriger Prävalenz der Hepatitis-B-Virus-(HBV-)Infektion in Deutschland ist es wichtig, vulnerable Gruppen und Ansatzpunkte für die Prävention zu identifizieren. In ersten Analysen der „Studie zur Gesundheit Erwachsener in Deutschland“ (DEGS1, 2008–2011) waren HBV-Infektion und -Impfung mit sozidemografischen Determinanten assoziiert. In dieser Arbeit werden die Ergebnisse im Detail untersucht.

**Material und Methoden:**

In DEGS1 lag für 7046 Teilnehmende (Alter: 18–79 Jahre) eine HBV-Serologie vor. Die stattgehabte HBV-Infektion war durch Antikörper gegen das Hepatitis-B-Core-Antigen (Anti-HBc) definiert, die impfinduzierte Immunität durch alleinigen Nachweis von Antikörpern gegen das Hepatitis-B-Surface-Antigen (Anti-HBs). Seroprävalenzen von HBV-Infektions- und -Impfstatus wurden geschlechtsstratifiziert geschätzt und Assoziationen mit Alter, Gemeindegröße, Einkommen, formaler Bildung, Krankenversicherung und Migrationsgeneration in logistischen Regressionen analysiert.

**Ergebnisse:**

Die HBV-Infektion war bei Männern und Frauen unabhängig mit den Altersgruppen 34–64 und ≥ 65 Jahre, erster Migrationsgeneration und Leben in größeren Gemeinden assoziiert, zudem bei Männern mit niedrigem Einkommen und bei Frauen mit niedriger Bildung. Die impfinduzierte Immunität war bei Männern und Frauen unabhängig mit den Altersgruppen 18–33 und 34–64 Jahre, mittlerer und hoher Bildung und hohem Einkommen assoziiert, darüber hinaus bei Männern mit mittlerem Einkommen und privater Krankenversicherung und bei Frauen mit fehlendem Migrationshintergrund.

**Diskussion:**

Die Berücksichtigung von Migrationsstatus, Einkommen und Bildung könnte zur zielgenauen Ausrichtung der HBV-Prävention beitragen.

**Zusatzmaterial online:**

Zusätzliche Informationen sind in der Online-Version dieses Artikels (10.1007/s00103-021-03473-z) enthalten.

## Abstract (en)

**Background and objective:**

Even though the prevalence of hepatitis B virus (HBV) infection in Germany is low, it is important to identify vulnerable groups and targeted approaches for infection prevention. Previous analyses from the “German Health Interview and Examination Survey for Adults” (DEGS1, 2008–2011) have shown that HBV infections and vaccination are associated with sociodemographic determinants. This paper examines the results in detail.

**Materials and methods:**

In the DEGS1, HBV serology was available for 7046 participants aged 18–79 years. HBV infection was defined by antibodies to hepatitis B core antigen (anti-HBc), vaccine-induced immunity by antibodies to hepatitis B surface antigen (anti-HBs) in the absence of other markers. Seroprevalences of HBV infection and vaccine-induced immunity were estimated stratified by sex, and associations with age, municipality size, income, formal education, health insurance and migration generation were analysed by logistic regression.

**Results:**

In both sexes, HBV infection was independently associated with age groups 34–64 and ≥ 65 years, first migrant generation and living in larger municipalities as well as low income in men and low education in women. Vaccine-induced immunity was independently associated with age groups 18–33 and 34–64 years, middle and high education and high income in both sexes, middle income and private health insurance in men and having no migration background in women.

**Conclusions:**

HBV prevention measures should take into account migration status, income and education in order to focus prevention measures.

## Einleitung

Die Hepatitis-B-Virus-(HBV-)Infektion ist eine der häufigsten Infektionskrankheiten weltweit, weist aber in der Prävalenz deutliche regionale Unterschiede auf [1]. Sie ist zudem ein bedeutender Risikofaktor für die Entwicklung einer Leberzirrhose und eines hepatozellulären Karzinoms und verursachte im Jahr 2015 weltweit ca. 887.000 Todesfälle [1]. Die Weltgesundheitsorganisation (WHO) hat ihren Mitgliedsstaaten daher zum Ziel gesetzt, die virale Hepatitis als Bedrohung für die öffentliche Gesundheit bis 2030 zu eliminieren. Eine 2016 verabschiedete Strategie sieht vor, dass Neuinfektionen und Mortalität durch weltweite synergistische Interventionen, wie z. B. eine Erhöhung der Impfquoten, eingedämmt werden [2]. Deutschlands nationale Strategie beinhaltet u. a. die Entwicklung von Präventions‑, Beratungs‑, Test- und Versorgungsangeboten, die sich an spezifischen Bedarfen von Risikogruppen, wie z. B. Jugendlichen, Migrantinnen und Migranten sowie Menschen mit beruflichem Expositionsrisiko, orientieren [3].

In der „Studie zur Gesundheit Erwachsener in Deutschland (DEGS1, 2008–2011)“ betrug die Prävalenz der aktiven (d. h. akuten oder chronischen) HBV-Infektion, definiert durch ein positives Hepatitis-B-Surface-Antigen (HBsAg), bei Erwachsenen im Alter von 18–79 Jahren 0,3 % [4]. Deutschland zählt damit zu den Niedrigprävalenzregionen, in denen die Infektion typischerweise im jungen Erwachsenenalter durch Risikoverhalten wie Drogengebrauch oder sexuelles Risikoverhalten erworben wird [5, 6]. Antikörper gegen das Hepatitis-B-Core-Antigen (Anti-HBc) stehen für eine stattgehabte oder aktive HBV-Infektion und waren bei 5,1 % der Allgemeinbevölkerung nachweisbar [4]. Verglichen mit den Ergebnissen des „Bundesgesundheitssurveys 98“ (BGS98, 1997–1999) war die Anti-HBc-Prävalenz von 8,7 % auf 5,1 % gesunken [4].

Die Prävention der HBV-Infektion beinhaltet z. B. das Screening von Personen, die Blut und Plasma spenden, die Förderung von Safer Sex und die Reduktion von Gefahren beim intravenösen Drogenkonsum [1]. Außerdem ist seit Anfang der 1980er-Jahre eine sichere und effektive Impfung gegen HBV verfügbar. Nachdem die Hepatitis-B-Impfung in Deutschland initial nur bei Risikogruppen wie medizinischem Personal oder Dialysepatienten eingesetzt wurde, empfahl die Ständige Impfkommission (STIKO) 1995 zusätzlich die Impfung von allen Kindern und Jugendlichen [7]. Die HBV-Impfung ist seitdem eine Standardimpfung für Säuglinge und wird bis zum 18. Geburtstag als Nachholimpfung angeboten [7]. In den bevölkerungsrepräsentativen Surveys BGS98 und DEGS1 stieg die Prävalenz der impfinduzierten Immunität, definiert durch alleinigen Nachweis von Antikörpern gegen das Hepatitis-B-Surface-Antigen (Anti-HBs), bei Erwachsenen im Alter von 18–79 Jahren von 4,3 % (1997–1999) auf 22,9 % (2008–2011; [4]). Querschnittsuntersuchungen in Risikogruppen zeigen jedoch eine erhöhte Prävalenz stattgehabter HBV-Infektionen bei gleichzeitig bestehenden Impflücken [8, 9]. Auch die für die Jahre 2008 bis 2011 errechnete Inzidenz von 0,9–1,0 gemeldeten Hepatitis-B-Fällen pro 100.000 Einwohnerinnen und Einwohner (EW) spricht für eine fortlaufende HBV-Transmission in Deutschland [10]. Eine zielgerichtete Prävention ist daher erforderlich, um besonders gefährdete Personen zu erreichen.

Übereinstimmend mit anderen bevölkerungsrepräsentativen Studien [11–15] ergab eine erste Analyse der DEGS1-Daten, dass die HBV-Durchseuchung mit dem Alter stieg und die Wahrscheinlichkeit für das Vorliegen einer impfinduzierten Immunität bei jüngeren Teilnehmenden und Frauen erhöht war [4]. Die HBV-Infektion war außerdem mit einem niedrigeren und die impfinduzierte Immunität mit einem höheren sozioökonomischen Status (SES) assoziiert, einem Index, der sich aus Einkommen, schulischer und beruflicher Bildung sowie beruflicher Stellung zusammensetzt [4, 16]. Internationale bevölkerungsbasierte Studien berichteten außerdem, dass HBV-Infektion und -Impfung mit Bildungsstand, Einkommen, Migrationsstatus und Gemeindegröße assoziiert waren [11–15, 17, 18]. Diese Determinanten wurden bislang in DEGS1 nicht differenziert untersucht; ihre Auswertung kann aber zusätzliche Erkenntnisse für die Verbesserung der HBV-Prävention liefern. Ziel dieser Studie war es daher, die Assoziationen der HBV-Infektion und -Impfung mit diesen Determinanten in der deutschen Allgemeinbevölkerung detailliert zu analysieren.

## Methoden

### Studiendesign

Die Zielpopulation von DEGS1 waren Erwachsene im Alter von 18–79 Jahren, die in Deutschland lebten und nicht in Institutionen, wie z. B. Flüchtlingsunterkünften, untergebracht waren [19].

Die Stichprobe umfasste ehemalige Teilnehmende des BGS98 (Response 64 %) und eine neu gezogene zweistufige, geschichtete Klumpenstichprobe (Response 42 %) [19]. In der neu gezogenen Stichprobe (*n* = 11.008 Personen) wurden 275 Personen aufgrund erheblicher Sprachbarrieren ausgeschlossen [19, 20]. Konzept und Design von DEGS1 sind an anderer Stelle ausführlich beschrieben [19–21]. Die Erhebungen fanden zwischen 2008 und 2011 an 180 Studienorten statt und umfassten u. a. Selbstausfüllfragebögen, Interviews und Blutuntersuchungen. Für Teilnehmende, die den Selbstausfüllfragebogen nicht vollständig bearbeiten konnten, wurde eine gekürzte Version vorgehalten. In der Kurzversion fehlten u. a. Fragen zu Krankenversicherung, Migrationsgeneration und Beruf. Fragebögen in weiteren Sprachen (Türkisch, Serbokroatisch, Russisch und Englisch) wurden Teilnehmenden angeboten, die zwar Deutsch sprechen, aber nicht ausreichend lesen und schreiben konnten.

### HBV-Serologie

Die Ergebnisse der HBV-Serologie wurden in 3 Kategorien unterteilt: 1) stattgehabte/aktive „HBV-Infektion“, definiert durch ein positives Anti-HBc, 2) „impfinduzierte Immunität“, definiert durch einen Anti-HBs-Spiegel ≥ 10 IU/l ohne Nachweis anderer HBV-Marker, und 3) „Suszeptibilität gegenüber HBV“, definiert durch das Fehlen von HBV-Markern (Tab. 1). Das serologische Testverfahren wurde an anderer Stelle ausführlich beschrieben [4].

**Table 1:** 

Ergebnisse der HBV-Serologie			Klinische Interpretation	Kategorie
Anti-HBc	Anti-HBs	HBsAg		
+	+	^a^	Erworbene Immunität gegen HBV nach einer Exposition	Stattgehabte HBV-Infektion
+	–	+	Aktuell vorliegende HBV-Infektion (akut oder chronisch)	
+	–	–	„Ausgeheilte“ oder okkulte HBV-Infektion (mit oder ohne Immunität gegen HBV und der Möglichkeit der Reaktivierung)	
–	+	–	Erworbene Immunität gegen HBV durch eine Impfung	Impfinduzierte Immunität gegen HBV
–	–	–	Kein Anhalt für eine stattgehabte Exposition gegenüber HBV oder eine Impfung	Suszeptibilität gegenüber HBV

Ergebnisse der HBV-Serologie: +: reaktiv; −: negativ

*HBV* Hepatitis-B-Virus

^a^Keine Untersuchung des HBsAg, wenn Anti-HBc und Anti-HBs reaktiv waren

### Unabhängige Variablen

Zur Schätzung der Prävalenzen wurden die Geburtsjahrgänge in 4‑Jahres-Kohorten unterteilt. Aufgrund kleiner Fallzahlen wurden die Geburtsjahrgänge 1989–1993 sowie 1928–1936 zusammengefasst. Für die logistische Regression wurden die Teilnehmenden in 3 Altersgruppen unterteilt: junge Erwachsene, die unter die Impfempfehlung für Kinder und Jugendliche fielen (18–33 Jahre), Erwachsene im mittleren Alter (34–64 Jahre) und Erwachsene im höheren Alter (≥ 65 Jahre).

Das Einkommen wurde als bedarfsgewichtetes Haushaltsnettoeinkommen (Nettoäquivalenzeinkommen) angegeben und fehlende Werte durch ein Regressionsmodell imputiert [16]. Das Einkommen wurde in 3 Gruppen unterteilt: ≤ 60 %, > 60 % bis 150 % und > 150 % des medianen Nettoäquivalenzeinkommens von privaten Haushalten in Deutschland [22]. Für den Studienzeitraum wurde für die Jahre 2007 bis 2010 ein Nettoäquivalenzeinkommen von 1558 €/Monat zugrunde gelegt [23] und die folgenden Kategorien wurden abgeleitet: „niedriges“ (≤ 935 €/Monat), „mittleres“ (936–2337 €/Monat) und „hohes Einkommen“ (≥ 2338 €/Monat).

Der formale Bildungsstand wurde in Anlehnung an die CASMIN-Klassifikation („Comparative Analyses of Social Mobility in Industrial Nations“) erfasst, die Schulbildung und berufliche Qualifikation berücksichtigt [16, 24]. Der Bildungsstand wurde nach Brauns et al. in 3 Kategorien unterteilt [25]: „niedrig“ (kein Abschluss, Hauptschulabschluss und/ohne berufliche Ausbildung), „mittel“ (Mittlere Reife, Fachhochschulreife oder Abitur, jeweils und/ohne berufliche Ausbildung) und „hoch“ (Fachhochschulabschluss, Hochschulabschluss).

Der Krankenversicherungsstatus war in „gesetzlich“, „privat“ und „andere“ kategorisiert. Personen der ersten Migrationsgeneration waren im Ausland geboren, während Personen der zweiten Migrationsgeneration in Deutschland geboren waren, aber mindestens einen im Ausland geborenen Elternteil hatten. Die Teilnehmenden sollten ihren aktuellen Beruf oder, falls sie nicht erwerbstätig waren, ihren zuletzt ausgeübten Beruf angeben. Die Kategorisierung der Berufe war an die „Klassifikation der Berufe 2010“ der Bundesagentur für Arbeit angelehnt [26]. Die für diese Analyse ausgewählten Berufe des Gesundheitswesens sind im Onlinematerial aufgeführt.

Die Gemeindegröße am 31.12.2006 wurde in ländlich (< 5000 EW), kleinstädtisch (5000 bis < 20.000 EW), mittelstädtisch (20.000 bis < 100.000 EW) und großstädtisch (≥ 100.000 EW) unterteilt.

### Statistische Auswertung

Die Auswertung erfolgte mit statistischen Verfahren für komplexe Stichproben in Stata/SE Version 14. Gewichtungsfaktoren korrigierten für Non-Response und Abweichungen von der Bevölkerungsstruktur in Hinblick auf Alter, Geschlecht, Region, Staatsangehörigkeit, Gemeindegröße und Bildungsstand [21]. Da Frauen häufiger eine impfinduzierte Immunität gegen HBV aufwiesen als Männer [4] und Risikofaktoren einer HBV-Infektion bei Männern und Frauen unterschiedlich verteilt sind [27, 28], wurden die Analysen nach Geschlecht stratifiziert. Die Prävalenzen der HBV-Infektion und impfinduzierten Immunität wurden als gewichtete Anteile in Prozent mit 95 %-Konfidenzintervallen (KI) geschätzt. Unterschiede wurden als statistisch signifikant angesehen, wenn sich die 95 %-Konfidenzintervalle nicht überlappten.

Assoziationen der HBV-Infektion und impfinduzierten Immunität mit Alter, Einkommen, Bildung, Krankenversicherung, Gemeindegröße und Migrationsgeneration wurden in univariabler und multivariabler logistischer Regression analysiert. Es wurden Odds Ratios (OR) und 95 %-Konfidenzintervalle geschätzt. Durch schrittweise Vorwärtsselektion wurde das am besten passende Modell für die jeweiligen Outcomes ermittelt. Ergebnisse mit einem *p*-Wert < 0,05 wurden als statistisch signifikant angesehen.

Analysen der DEGS1-Daten haben gezeigt, dass bei Teilnehmenden mit Migrationshintergrund trotz des Einsatzes von Gewichtungsfaktoren keine Repräsentativität für Alter und Migrationsgeneration vorlag [29, 30]. Analysen von Teilnehmenden mit Migrationshintergrund sollten deshalb nach Alter, Geschlecht, Migrationsgeneration und sozioökonomischem Status stratifiziert werden [29, 30]. Die Variable „Migrationsgeneration“ wurde daher nur in multivariablen Analysen untersucht.

Da Informationen zu einer „aktuellen/früheren Berufstätigkeit im Gesundheitswesen“ nur bei Teilnehmenden erhoben wurde, die jemals erwerbstätig gewesen waren, war der Anteil fehlender Angaben relativ hoch (Tab. 2). Auch in der Kategorie „fehlende/andere Krankenversicherung“ waren die Fallzahlen niedrig (Tab. 2), sodass die Variable „Beruf“ und die Kategorie „fehlende/andere Krankenversicherung“ in den logistischen Regressionen nicht berücksichtigt wurden.

**Table 2:** 

	Männer		Frauen	
	Anzahl^a^	Gewichtete Anteile in % (95 %-KI)	Anzahl^a^	Gewichtete Anteile in % (95 %-KI)
*Studienpopulation*	3376	–	–	–
*Alter in Jahren*				
18–33	657	24,5 (22,8–26,3)	694	23,9 (22,3–25,6)
34–64	1805	57,4 (55,5–59,2)	2062	55,3 (53,4–57,2)
≥ 65	914	18,1 (17,0–19,4)	914	20,8 (19,3–22,3)
Fehlt	0	0	0	0
*Gemeindegröße*				
Ländlich	651	16,0 (10,9–23,0)	642	14,7 (9,8–21,4)
Kleinstädtisch	832	23,9 (17,9–31,3)	875	24,1 (17,9–31,5)
Mittelstädtisch	958	28,8 (22,1–36,5)	1086	29,7 (22,9–37,5)
Großstädtisch	935	31,3 (24,4–39,1)	1067	31,6 (24,7–39,5)
Fehlt	0	0	0	0
*Einkommen*				
Niedrig	929	30,1 (27,8–32,6)	1075	32,2 (30,2–34,2)
Mittel	1979	57,3 (54,7–59,9)	2199	58,1 (56,0–60,2)
Hoch	468	12,6 (11,0–14,4)	396	9,8 (8,4–11,3)
Fehlt	0	0	0	0
*Bildungsstand*				
Niedrig	1096	36,1 (33,3–39,0)	1195	37,4 (35,0–39,9)
Mittel	1502	46,5 (44,1–48,9)	1889	49,6 (47,3–51,9)
Hoch	754	16,5 (14,9–18,4)	563	12,0 (10,4–13,9)
Fehlt	24	0,9 (0,5–1,5)	23	1,0 (0,6–1,8)
*Krankenversicherung*				
Gesetzlich	2765	81,9 (80,0–83,7)	3251	88,8 (87,3–90,2)
Privat	492	14,0 (12,5–15,8)	348	8,6 (7,4–10,1)
Fehlt/andere	119	4,1 (3,2–5,2)	71	2,5 (1,9–3,4)
*Migrationsgeneration*				
Keine	2783	77,3 (74,4–79,9)	3037	76,6 (74,1–79,0)
Erste	277	12,2 (10,3–14,3)	321	13,2 (11,3–15,4)
Zweite und weitere	198	6,5 (5,4–7,9)	205	6,3 (5,2–7,6)
Fehlt	118	4,0 (3,1–5,1)	107	3,9 (3,0–5,0)
*Letzter/aktueller Beruf*^b^				
Gesundheitsberuf	59	2,2 (1,6–3,1)	344	9,9 (8,7–11,4)
Anderer	2900	89,4 (87,3–91,2)	2899	82,1 (80,2–83,8)
Fehlt	226	8,4 (6,8–10,3)	222	8,0 (6,6–9,6)

Die Ergebnisse werden als ungewichtete Fallzahlen und gewichtete Anteile mit 95%-Konfidenzintervallen (KI) berichtet. Gewichtungsfaktoren wurden verwendet, um für Non-Response und Abweichungen von der Zielpopulation in Bezug auf Alter, Geschlecht, Region, Staatsangehörigkeit, Gemeindegröße und Bildungsstand zu korrigieren [19]. *n*_ungewichtet_ = 7046

^a^ Ungewichtete Anzahl

^b^ Nur verfügbar bei Teilnehmenden, die jemals erwerbstätig waren (Männer: *n* = 3185, Frauen: *n* = 3465)

## Ergebnisse

Insgesamt hatten 7115 Erwachsene im Alter von 18–79 Jahren die Befragung und den Untersuchungsteil absolviert. Ergebnisse der HBV-Serologie lagen für 7046 Teilnehmende (99 %) vor. Die soziodemografischen Charakteristika dieser Teilnehmenden sind in Tab. 2 dargestellt.

### HBV-Infektion

Die *Prävalenz* der HBV-Infektion betrug 5,3 % (95 %-KI 4,3–6,5) bei Männern und 4,8 % (95 %-KI 4,0–5,8) bei Frauen.

**Figure 1:**
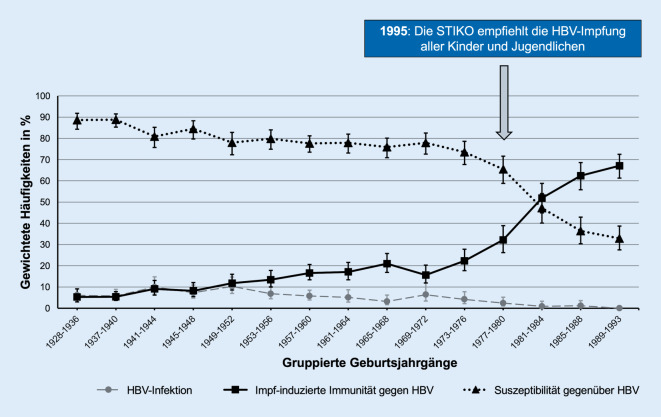

Teilnehmende der Geburtsjahrgänge 1949–1952 wiesen die höchste Prävalenz von HBV-Infektionen auf (10,3 %; 95 %-KI 7,0–14,7), während in den Geburtsjahrgängen 1989–1993 keine stattgehabten HBV-Infektionen nachgewiesen wurden (Abb. 1).

Bei Männern und Frauen waren die Prävalenzen von HBV-Infektionen am höchsten in der Altersgruppe ≥ 65 Jahre sowie bei Teilnehmenden mit niedrigem Einkommen und niedriger Bildung (Tab. 3). Bezogen auf die Gemeindegröße waren die Prävalenzen von HBV-Infektionen bei Männern in mittelgroßen Städten und bei Frauen in Großstädten am höchsten. Die Prävalenz von HBV-Infektionen unterschied sich nicht zwischen den im Gesundheitswesen beschäftigten Teilnehmenden und jenen ohne eine solche Berufstätigkeit (Tab. 4).

**Table 3:** 

	Männer					Frauen				
	Gewichtete Prävalenz^a^ der HBV-Infektion	Univariable logistische Regression		Multivariable logistische Regression (*n* = 3258)		Gewichtete Prävalenz^a^ der HBV-Infektion	Univariable logistische Regression		Multivariable logistische Regression (*n* = 3561)	
	% (95 %-KI)	Odds Ratio (95 %-KI)	*p*-Wert^b^	Adjustierte^c^ Odds Ratio (95 %-KI)	*p*-Wert^b^	% (95 %-KI)	Odds Ratio (95 %-KI)	*p*-Wert^b^	Adjustierte^c^ Odds Ratio (95 %-KI)	*p*-Wert^b^
*Insgesamt*										
	5,3 (4,3–6,5)	–	–	–	–	4,8 (4,0–5,8)	–	–	–	–
*Alter in Jahren*										
18–33	1,6 (0,8–3,1)	Referenz	–	Referenz	–	1,0 (0,3–2,9)	Referenz	–	Referenz	–
34–64	6,0 (4,6–7,8)	3,95 (1,88–8,33)	*	5,10 (2,32–11,20)	*	6,0 (4,7–7,6)	6,36 (2,07–19,59)	0,001	8,16 (2,75–24,17)	*
≥ 65	8,3 (5,9–11,5)	5,58 (2,59–12,05)	*	8,19 (3,63–18,49)	*	6,1 (4,3–8,5)	6,42 (2,03–20,35)	0,002	11,20 (3,63–34,52)	*
*Gemeindegröße*										
Ländlich	2,7 (1,6–4,4)	Referenz	–	Referenz	–	2,7 (1,6–4,6)	Referenz	–	Referenz	–
Kleinst.	3,9 (2,5–6,0)	1,49 (0,74–2,99)	0,259	1,20 (0,59–2,44)	0,605	4,3 (2,8–6,6)	1,63 (0,79–3,34)	0,184	0,80 (0,37–1,74)	0,575
Mittelst.	7,4 (5,3–10,1)	2,92 (1,56–5,47)	0,001	2,32 (1,20–4,47)	0,012	4,5 (3,1–6,3)	1,69 (0,86–3,30)	0,127	1,22 (0,63–2,37)	0,554
Großst.	5,9 (4,1–8,4)	2,31 (1,22–4,39)	0,011	1,78 (0,88–3,58)	0,108	6,6 (4,9–8,7)	2,53 (1,33–4,79)	0,005	2,01 (1,05–3,84)	0,034
*Einkommen*										
Niedrig	8,6 (6,3–11,8)	3,35 (1,86–6,03)	*	2,59 (1,38–4,86)	0,003	6,6 (4,8–9,0)	3,83 (1,50–9,79)	0,005	–	–
Mittel	4,2 (3,0–5,6)	1,54 (0,81–2,92)	0,183	1,37 (0,70–2,68)	0,363	4,4 (3,4–5,6)	2,47 (1,03–5,93)	0,044	–	–
Hoch	2,7 (1,6–4,6)	Referenz	–	Referenz	–	1,8 (0,8–4,1)	Referenz	–	–	–
*Bildungsstand*										
Niedrig	7,0 (5,2–9,4)	1,37 (0,84–2,24)	0,205	–	–	7,7 (5,9–9,9)	3,00 (1,56–5,77)	0,001	2,24 (1,10–4,58)	0,027
Mittel	4,1 (3,0–5,7)	0,78 (0,48–1,28)	0,321	–	–	3,1 (2,3–4,3)	1,16 (0,57–2,34)	0,681	1,72 (0,83–3,55)	0,143
Hoch	5,2 (3,7–7,4)	Referenz	–	–	–	2,7 (1,5–4,9)	Referenz	–	Referenz	–
*Krankenversicherung*										
GKV^d^	5,6 (4,5–6,8)	1,56 (0,81–3,01)	0,180	–	–	4,6 (3,7–5,6)	2,30 (0,82–6,49)	0,114	–	–
PKV^d^	3,6 (1,9–6,8)	Referenz	–	–	–	2,0 (0,7–5,5)	Referenz	–	–	–
*Migrationsgeneration*^*e*^										
Keine	–	–	–	Referenz	–	–	–	–	Referenz	–
Erste	–	–	–	6,03 (3,70–9,81)	*	–	–	–	7,26 (4,37–12,07)	*
Zweite	–	–	–	0,57 (0,19–1,64)	0,292	–	–	–	1,57 (0,63–3,96)	0,333

In allen Analysen wurde nach Geschlecht stratifiziert, *n*_ungewichtet gesamt_ = 7046. *p-Wert < 0,001

^a^ Zur Schätzung der Prävalenzen wurden Gewichtungsfaktoren eingesetzt, um für Non-Response und für Abweichungen von der Zielpopulation in Bezug auf Alter, Geschlecht, Region, Staatsangehörigkeit, Gemeindegröße und Bildungsstand zu korrigieren [19]

^b^ Ergebnisse mit einem *p*-Wert < 0,05 wurden als statistisch signifikant angesehen

^c^ Das am besten passende Modell wurde durch schrittweise Vorwärtsselektion ermittelt

^d^ *GKV* gesetzliche Krankenversicherung, *PKV* private Krankenversicherung, *Kleinst.* kleinstädtisch, *Mittelst.* mittelstädtisch, *Großst.* großstädtisch

^e^ Aufgrund der eingeschränkten Repräsentativität wurde die Variable „Migrationsgeneration“ nur in der multivariablen logistischen Regression untersucht

**Table 4:** 

	Beruf im Gesundheitswesen *n* = 403		Andere Berufe *n* = 5799	
	Anzahl	Gewichtete Prävalenz in % (95 %-KI)	Anzahl	Gewichtete Prävalenz in % (95 %-KI)
HBV-Infektion	21	4,1 (2,5–6,7)	234	4,3 (3,6–5,1)
Impfinduzierte Immunität gegen HBV	266	69,1 (62,8–74,7)	1022	18,6 (17,2–20,2)
Suszeptibilität gegenüber HBV	116	26,9 (21,4–33,2)	4543	77,1 (75,4–78,8)

Einen Überblick über die eingeschlossenen „Berufe im Gesundheitswesen“ gibt Onlinematerial 1. Die Ergebnisse werden als ungewichtete Fallzahlen und gewichtete Prävalenzen mit 95-%-Konfidenzintervall (KI) berichtet. Gewichtungsfaktoren wurden eingesetzt, um für Non-Response und für Abweichungen von der Zielpopulation in Bezug auf Alter, Geschlecht, Region, Staatsangehörigkeit, Gemeindegröße und Bildungsstand zu korrigieren [19]. *n*_ungewichtet gesamt_ = 6202

In den *multivariablen Analysen* war eine HBV-Infektion bei Männern und Frauen statistisch signifikant mit höherer Altersgruppe (34–64, ≥ 65 Jahre) und Zugehörigkeit zur ersten Migrationsgeneration assoziiert (Tab. 3). Die HBV-Infektion war außerdem mit niedrigem Einkommen (Männer) bzw. niedriger Bildung (Frauen) assoziiert. Darüber hinaus zeigte sich eine statistisch signifikante Assoziation der HBV-Infektion mit dem Leben in mittelgroßen Städten (Männer) bzw. in Großstädten (Frauen).

### Impfinduzierte Immunität

Die *Prävalenz* der impfinduzierten Immunität gegen HBV war bei Frauen signifikant höher (25,8 %; 95 %-KI 23,8–27,8) als bei Männern (20,1 %; 95 %-KI 18,2–22,1).

Die Prävalenz der impfinduzierten Immunität war am niedrigsten bei den Geburtsjahrgängen 1928–1936 (6,1 %; 95 %-KI 4,1–9,2) und am höchsten bei den Geburtsjahrgängen 1989–1993 (67,1 %; 95 %-KI 61,3–72,5; Abb. 1). Die Geburtsjahrgänge 1981–1984, die als erste unter die allgemeine Impfempfehlung für Kinder und Jugendliche gefallen waren, wiesen eine signifikant höhere Prävalenz der impfinduzierten Immunität auf als die vorhergehenden Geburtsjahrgänge 1977–1980 (51,9 %; 95 %-KI 44,9–58,8 vs. 32,2 %; 95 %-KI 26,2–38,9).

Bei Männern und Frauen fanden sich die höchsten Prävalenzen der impfinduzierten Immunität in der Altersgruppe 18–33 Jahre sowie bei Teilnehmenden mit hohem Einkommen. Die höchsten Prävalenzen impfinduzierter Immunität zeigten sich außerdem bei Männern mit mittlerer und bei Frauen mit hoher Bildung (Tab. 5). Männer mit privater Krankenversicherung (PKV) wiesen signifikant häufiger eine impfinduzierte Immunität auf als Männer mit gesetzlicher Krankenversicherung (GKV). Bei Teilnehmenden mit einem Beruf im Gesundheitswesen war die Prävalenz der impfinduzierten Immunität signifikant höher als bei Teilnehmenden mit anderen Berufen (Tab. 4).

**Table 5:** 

	Männer					Frauen				
	Gewichtete Prävalenz^a^ der impfinduzierten Immunität gegen HBV	Univariable logistische Regression		Multivariable logistische Regression (*n* = 3258)		Gewichtete Prävalenz^a^ der impfinduzierten Immunität gegen HBV	Univariable logistische Regression		Multivariable logistische Regression (*n* = 3561)	
	% (95 %-KI)	Odds Ratio (95 %-KI)	*p*-Wert^b^	Adjustierte^c^ Odds Ratio (95 %-KI)	*p*-Wert^b^	% (95 %-KI)	Odds Ratio (95 %-KI)	*p*-Wert^b^	Adjustierte^c^ Odds Ratio (95 %-KI)	*p*-Wert^b^
*Insgesamt*										
	20,1 (18,2–22,1)	–	–	–	–	25,8 (23,8–27,8)	–	–	–	–
*Alter in Jahren*										
18–33	48,1 (43,7–52,5)	16,96 (10,70–26,87)	*	18,08 (10,32–31,69)	*	56,3 (51,7–60,8)	17,38 (11,98–25,23)	*	14,85 (9,80–22,51)	*
34–64	12,8 (10,9–15,0)	2,69 (1,74–4,16)	*	2,20 (1,37–3,54)	0,001	19,7 (17,3–22,3)	3,31 (2,35–4,65)	*	2,78 (1,89–4,09)	*
≥65	5,2 (3,4–7,7)	Referenz	–	–	–	6,9 (5,1–9,4)	Referenz–	–	Referenz	–
*Gemeindegröße*										
Ländlich	18,8 (14,5–24,0)	Referenz	–	–	–	23,0 (19,9–26,5)	Referenz–	–	–	–
Kleinst.	19,9 (16,2–24,1)	1,07 (0,72–1,60)	0,724	–	–	26,4 (22,3–31,0)	1,20 (0,89–1,61)	0,226	–	–
Mittelst.	21,3 (17,8–25,2)	1,17 (0,80–1,71)	0,423	–	–	26,6 (23,2–30,4)	1,21 (0,93–1,58)	0,151	–	–
Großst.	19,8 (16,8–23,3)	1,07 (0,74–1,55)	0,719	–	–	25,8 (22,2–29,7)	1,16 (0,88–1,52)	0,280	–	–
*Einkommen*										
Niedrig	17,0 (14,2–20,3)	Referenz	–	Referenz	–	24,4 (21,3–27,8)	Referenz	–	Referenz	–
Mittel	18,9 (16,3–21,7)	1,13 (0,86–1,49)	0,381	1,38 (1,01–1,89)	0,047	25,6 (23,0–28,3)	1,06 (0,85–1,33)	0,581	1,22 (0,96–1,55)	0,109
Hoch	32,9 (28,0–38,3)	2,39 (1,69–3,37)	*	2,94 (1,91–4,52)	*	31,6 (26,2–37,6)	1,43 (1,05–1,96)	0,026	1,44 (1,02–2,03)	0,040
*Bildungsstand*										
Niedrig	10,2 (8,2–12,7)	Referenz	–	Referenz	–	12,2 (9,9–14,9)	Referenz	–	Referenz	–
Mittel	26,3 (23,5–29,3)	3,13 (2,39–4,09)	*	1,57 (1,15–2,14)	0,005	33,8 (30,9–36,7)	3,67 (2,82–4,77)	*	2,12 (1,58–2,86)	*
Hoch	24,6 (20,3–29,5)	2,86 (1,99–4,10)	*	1,70 (1,13–2,56)	0,011	35,6 (29,9–41,7)	3,98 (2,78–5,68)	*	2,25 (1,49–3,40)	*
*Krankenversicherung*										
GKV^d^	18,4 (16,4–20,5)	Referenz	–	Referenz	–	–	Referenz	–	–	–
PKV^d^	29,7 (24,8–35,0)	1,88 (1,42–2,48)	*	1,91 (1,38–2,64)	*	26,0 (20,4–32,4)	0,99 (0,70–1,40)	0,964	–	–
*Migrationsgeneration*^*e*^										
Keine	–	–	–	Referenz	–	–	–	–	Referenz	–
Erste	–	–	–	0,77 (0,48–1,23)	0,269	–	–	–	0,60 (0,41–0,88)	0,009
Zweite	–	–	–	0,66 (0,40–1,08)	0,098	–	–	–	0,66 (0,43–1,02)	0,061

In allen Analysen wurde nach Geschlecht stratifiziert, *n*_ungewichtet gesamt_ = 7046. * *p*-Wert < 0,001

^a^ Zur Schätzung der Prävalenzen wurden Gewichtungsfaktoren eingesetzt, um für Non-Response und für Abweichungen von der Zielpopulation in Bezug auf Alter, Geschlecht, Region, Staatsangehörigkeit, Gemeindegröße und Bildungsstand zu korrigieren [19]

^b^ Ergebnisse mit einem *p*-Wert < 0,05 wurden als statistisch signifikant angesehen

^c^ Das am besten passende Modell wurde durch schrittweise Vorwärtsselektion ermittelt

^d^ *GKV* gesetzliche Krankenversicherung, *PKV* private Krankenversicherung, *Kleinst.* kleinstädtisch, *Mittelst.* mittelstädtisch, *Großst.* großstädtisch

^e^ Aufgrund der eingeschränkten Repräsentativität wurde die Variable „Migrationsgeneration“ nur in der multivariablen logistischen Regression untersucht

In den *multivariablen Analysen* für Männer und Frauen war die impfinduzierte Immunität statistisch signifikant mit jüngerer Altersgruppe (18–33, 34–64 Jahre), mittlerer und hoher Bildung sowie hohem Einkommen assoziiert (Tab. 5). Bei Männern zeigte sich außerdem eine statistisch signifikante Assoziation der impfinduzierten Immunität mit mittlerem Einkommen und privater Krankenversicherung. Die Chance, dass Frauen der ersten Migrationsgeneration eine impfinduzierte Immunität hatten, war signifikant geringer als bei Frauen ohne Migrationshintergrund.

### Suszeptibilität

Bei Männern war der Anteil suszeptibler Personen signifikant höher (74,6 %; 95 %-KI 72,4–76,7) als bei Frauen (69,4 %; 95 %-KI 67,3–71,4). Die höchste Prävalenz suszeptibler Personen fand sich in den Geburtsjahrgängen 1937–1940, die niedrigste in den Geburtsjahrgängen 1989–1993 (88,8 %; 95 %-KI 85,3–91,5 vs. 32,9 %; 95 %-KI 27,5–38,7; Abb. 1).

## Diskussion

Basierend auf einem bevölkerungsrepräsentativen Survey aus Deutschland, einem Land mit niedriger HBsAg-Prävalenz, wurden in dieser Arbeit mögliche soziodemografische Determinanten der HBV-Infektion und impfinduzierten Immunität bei Männern und Frauen im Alter von 18–79 Jahren untersucht. In dieser Querschnittuntersuchung zeigte sich eine gegensätzliche Assoziation von stattgehabter HBV-Infektion und impfinduzierter Immunität mit den meisten der untersuchten Determinanten: Während die stattgehabte HBV-Infektion mit niedrigem Einkommen (Männer) und niedriger Bildung (Frauen) assoziiert war, waren höheres Einkommen und Bildung wichtige Determinanten der impfinduzierten Immunität bei beiden Geschlechtern. Teilnehmende der ersten Migrationsgeneration hatten eine signifikant erhöhte Chance für das Vorliegen einer stattgehabten HBV-Infektion, gleichzeitig war die Chance für das Vorliegen einer impfinduzierten Immunität bei Frauen der ersten Migrationsgeneration signifikant geringer als bei Frauen ohne Migrationshintergrund.

Im Jahr 1995 wurde in Deutschland die allgemeine Impfempfehlung für Kinder und Jugendliche ausgesprochen. Bereits die Geburtsjahrgänge (1981–1984) wiesen eine signifikant höhere Prävalenz der impfinduzierten Immunität auf als die vorhergehenden Geburtsjahrgänge 1977–1980, die zum Zeitpunkt der Impfempfehlung bereits im Jugendalter waren. Allerdings lag die Prävalenz der impfinduzierten Immunität bei den späteren Geburtsjahrgängen (1989–1993) mit maximal 67,1 % weiterhin deutlich unter dem von der WHO definierten Impfquotenziel von ≥ 95 % [2]. Möglicherweise ist dies dadurch zu erklären, dass auch die Teilnehmenden dieser Jahrgänge zum Zeitpunkt der Impfempfehlung 1995 bereits älter waren als der im Routineimpfschema für die Grundimmunisierung von Kindern angegebene Altersbereich. Bei ihnen hätte eine HBV-Impfung daher als Nachholimpfung (Catch-up-Impfung) durchgeführt werden müssen. Es ist bekannt, dass Nachholimpfungen seltener in Anspruch genommen werden als Standardimpfungen, die im Rahmen eines aktuell für das jeweilige Alter gültigen Impfkalenders angeboten werden [31].

Wie bereits in anderen bevölkerungsbasierten Surveys war auch in unserer Studie die Zugehörigkeit zur ersten Migrationsgeneration ein bedeutender Faktor für eine stattgehabte HBV-Infektion [11–15]. In einer französischen Studie variierte das Risiko einer HBV-Infektion bei Menschen der ersten Migrationsgeneration mit der HBsAg-Prävalenz im Herkunftsland [12]. Die erhobenen Angaben zu den Herkunftsländern der Teilnehmenden in DEGS1 waren aus methodischen Gründen nicht auswertbar. Aufgrund einer Analyse der Staatsangehörigkeiten der Teilnehmenden [30] ist jedoch davon auszugehen, dass die Stichprobe Teilnehmende aus Ländern mit niedriger, mittlerer und hoher HBsAg-Prävalenz enthielt. Das Risiko einer HBV-Infektion wurde daher vermutlich für Menschen aus Ländern mit hoher HBsAg-Prävalenz unterschätzt bzw. für Menschen aus Ländern mit niedriger HBsAg-Prävalenz überschätzt.

Trotz einer niedrigen HBsAg-Prävalenz im Aufnahmeland haben Menschen der ersten Migrationsgeneration auch hier in Deutschland ein erhöhtes Risiko, sich mit HBV zu infizieren, da meist enge Kontakte zu anderen Menschen mit Migrationserfahrung und dem Herkunftsland bestehen [14, 32, 33]. Sie sind daher eine wichtige Zielgruppe der HBV-Impfung. Der Zugang zu Präventionsmaßnahmen kann für Menschen der ersten Migrationsgeneration aufgrund von sprachlichen und kulturellen Barrieren erschwert sein [34]. So ergab eine frühere Auswertung des DEGS1, dass Frauen der ersten Migrationsgeneration über präventive Gesundheitsangebote weniger informiert waren und diese auch seltener in Anspruch nahmen als Frauen ohne Migrationshintergrund [29]. Da auch in unseren Auswertungen Frauen der ersten Migrationsgeneration eine geringere Chance für eine impfinduzierte Immunität hatten als Frauen ohne Migrationshintergrund, sollten die besonderen Bedarfe des Zugangs zu Impfung und Prävention für diese Gruppe weiter untersucht und entsprechende Maßnahmen umgesetzt werden.

In den über schrittweise Vorwärtsselektion gebildeten multivariablen Analysen, in der alle in univariablen Analysen identifizierten Faktoren untersucht wurden, konnte eine Assoziation der HBV-Infektion bei Männern unabhängig mit einem niedrigen Einkommen und bei Frauen unabhängig mit einer niedrigen Bildung beobachtet werden. Da Einkommen, Bildung und Migrationsstatus eng miteinander korrelieren, haben wir in zusätzlichen explorativen, multivariablen Analysen differenziert für Männer und Frauen Interaktionsterme eingeschlossen (Ergebnisse nicht gezeigt). Frauen mit niedrigem Einkommen und niedriger Bildung hatten – anders als Männer – geringfügig höhere Chancen einer HBV-Infektion. Eine weitere Interaktion konnte für Frauen zwischen der ersten Einwanderungsgeneration und niedriger Bildung sowie für Männer zwischen der ersten Einwanderungsgeneration und niedrigem Einkommen festgestellt werden.

In anderen bevölkerungsbasierten Studien war die stattgehabte HBV-Infektion ebenfalls mit einem niedrigen Einkommen bzw. materieller Armut assoziiert [12, 15] und zeigte widersprüchliche Ergebnisse bezüglich der Assoziation mit dem Bildungsstand [12, 15]. Allerdings war ein niedriger sozioökonomischer Status in einer französischen Studie auch mit weiteren Risikofaktoren der HBV-Infektion, wie der Herkunft aus Ländern mit erhöhter HBsAg-Prävalenz, Tätowierungen, Drogengebrauch oder früherer Inhaftierung, assoziiert [12]. Ein Confounding durch Einflussgrößen, die in DEGS1 nicht erfasst wurden, ist somit nicht auszuschließen.

Internationale Studien zeigen, dass ein höherer Bildungsstand mit einem höheren Wissensstand zur HBV-Infektion, deren Risiken und Präventionsmaßnahmen einhergeht [12, 35, 36]. In unserer Studie war die impfinduzierte Immunität bei beiden Geschlechtern unabhängig mit mittlerer und hoher Bildung assoziiert und bestätigte die Ergebnisse anderer Untersuchungen [15, 37, 38].

Auch ein hohes Einkommen war bei Männern und Frauen unabhängig mit dem Vorliegen einer impfinduzierten Immunität assoziiert. Eine mögliche Erklärung hierfür könnten die Regelungen für eine Kostenübernahme der Impfungen sein: Grundsätzlich werden in Deutschland alle durch die STIKO empfohlenen Impfungen von der GKV übernommen [39]. Ausnahmen sind Impfungen aufgrund eines berufsassoziierten Risikos, die vom Arbeitgeber bezahlt werden, und Impfungen in Vorbereitung privater (Fern‑)Reisen [7]. Diese mussten bis 2007 stets selbst bezahlt werden [7]. Da in einem Telefon-Survey und in einer Studie unter Blutspendenden „private Reisen“ als eine der Hauptindikationen für eine Hepatitis-B-Impfung genannt wurden [37, 38], könnte ein hoher Anteil an Reiseimpfungen die Assoziation der impfinduzierten Immunität mit höherem Einkommen erklären.

Eine private Krankenversicherung bietet umfangreichere Leistungspakete und höhere Honorare für Ärztinnen und Ärzte als die GKV, setzt bei Angestellten aber das Überschreiten einer bestimmten Einkommensgrenze voraus [39]. 2012 waren 11 % der deutschen Bevölkerung privat krankenversichert [39]. In dieser Studie war die Chance für das Vorliegen einer impfinduzierten Immunität bei Männern mit privater Krankenversicherung fast doppelt so hoch wie bei Männern mit gesetzlicher Krankenversicherung. Bei Frauen hingegen fand sich diese Assoziation nicht; allerdings waren Frauen auch seltener privat krankenversichert. Da die Frage der Kostenerstattung nach Angabe von befragten Ärztinnen und Ärzten eine mögliche Barriere für Impfungen ist [40], könnte die Zugehörigkeit zur PKV das Angebot und die Inanspruchnahme der HBV-Impfung begünstigen.

Das Risiko einer HBV-Infektion ist bei medizinischem Personal erhöht, z. B. durch häufigen Kontakt zu Körperflüssigkeiten und Stichverletzungen. Für Gesundheitspersonal in Deutschland besteht deshalb seit Beginn der 1980er-Jahre eine HBV-Impfempfehlung einschließlich einer Auffrischungsimpfung bei sinkendem Anti-HBs-Titer [41], sodass neue Meldungen berufsassoziierter HBV-Infektionen heutzutage selten sind [42].

In dieser Studie untersuchten wir ausgewählte medizinische Berufsgruppen, die direkten Patientenkontakt und ein erhöhtes Risiko für eine Exposition gegenüber potenziell infektiösen Körperflüssigkeiten haben. Teilnehmende mit einem Beruf im Gesundheitswesen wiesen keine höhere Prävalenz stattgehabter HBV-Infektionen auf als Teilnehmende ohne medizinischen Beruf. Die Prävalenz der impfinduzierten Immunität war hingegen bei Teilnehmenden mit einem Beruf im Gesundheitswesen signifikant höher. Interessanterweise wiesen ca. 30 % der Teilnehmenden mit einem medizinischen Beruf keine impfinduzierte Immunität gegen HBV auf und fast 27 % waren für eine HBV-Infektion suszeptibel. Hierbei ist zu berücksichtigen, dass die Indikation für eine HBV-Impfung immer auf einer individuellen, arbeitsplatzbezogenen Risikobeurteilung beruht. In bestimmten Fällen (wie z. B. bei einer reinen Verwaltungstätigkeit) mag daher auch bei Personen mit medizinischen Berufen keine Indikation zur HBV-Impfung bestehen. Auch ist die HBV-Impfung für Gesundheitspersonal nicht verpflichtend.

### Stärken und Limitationen

Die Repräsentativität der DEGS1-Daten für die deutsche Allgemeinbevölkerung ist hoch und die Teilnahmerate der neu gezogenen Stichprobe mit anderen europäischen Surveys vergleichbar [19, 43]. Allerdings liegt der Beginn der Datenerhebung bereits mehr als 10 Jahre zurück. Obwohl die Daten aus DEGS1 absehbar noch für einige Jahre die aktuellste bevölkerungsbasierte Datenbasis für die untersuchten Fragestellungen darstellen, können sich die aktuellen Prävalenzen und Zusammenhänge in der Zwischenzeit verändert haben.

Zur Minimierung möglicher Verzerrungen durch selektives Teilnahmeverhalten kamen Non-Responder-Analysen, Vergleiche mit der Zielpopulation und Gewichtungsfaktoren zum Einsatz [19, 20]. In DEGS1 wurden allerdings nur Personen eingeschlossen, die außerhalb von Institutionen, wie z. B. Flüchtlingseinrichtungen oder Gefängnissen, lebten [19]. Bestimmte Risikogruppen der HBV-Infektion, wie Inhaftierte oder Flüchtlinge [1, 44], wurden daher nicht untersucht. Auch waren die Auswertungen für Menschen mit Migrationshintergrund nur mit Einschränkungen möglich [29, 30].

Das Vorliegen einer impfinduzierten Immunität bzw. einer stattgehabten HBV-Infektion wurde serologisch ermittelt. Es wird angenommen, dass ein Anti-HBs-Titer von > 10 IU/l einen langfristigen Schutz vor einer Hepatitis B bietet. Allerdings sprechen ca. 5 % der gesunden Erwachsenen nicht auf die Hepatitis-B-Impfung an und auch nach erfolgreicher Impfung fällt der Anti-HBs-Titer nach 4–10 Jahren bei bis zu 50 % der gesunden Erwachsenen auf niedrige oder nicht mehr nachweisbare Spiegel [45–49]. Die tatsächliche Impfquote wurde daher vermutlich in dieser Studie unterschätzt. Hierfür spricht auch, dass laut Auswertung der Daten auf Basis der vorgelegten Impfbücher und der Selbstauskünfte 32,9 % der DEGS1-Teilnehmenden mindestens eine Hepatitis-B-Impfung erhalten hatten [50], aber nur 22,9 % eine serologisch nachweisbare impfinduzierte Immunität aufwiesen [4].

In dieser Studie werden stattgehabte HBV-Infektionen, die z. T. bereits vor Jahrzehnten und/oder außerhalb Deutschlands erworben wurden, als Zeichen für ein aktuell erhöhtes Infektionsrisiko verwendet. Da sich z. B. die soziodemografischen Merkmale der Betroffenen seit der Infektion verändert haben könnten, sollte dies bei der Interpretation der Ergebnisse berücksichtigt werden.

## Fazit

Obwohl die zugrunde liegenden Daten bereits vor rund einer Dekade erhoben wurden, können die Ergebnisse dieser Studie dazu beitragen, die HBV-Prävention in Deutschland zielgenauer auszurichten.

Zwar legt die steigende Prävalenz der impfinduzierten Immunität in den jüngeren Altersgruppen nahe, dass die Impfung die HBV-Transmission in der Zukunft begrenzen wird. Um das Ziel der Elimination der Virushepatitis bis 2030 zu erreichen, sind jedoch weitere Maßnahmen erforderlich, z. B. um Impflücken zu schließen.

Die Ergebnisse dieser Studie liefern Hinweise, dass Präventionsstrategien insbesondere die Bedarfe von Menschen mit niedrigem Bildungsstand und eigener Migrationserfahrung berücksichtigen sollten. Auch könnten evtl. die Regelungen der Kostenübernahme der HBV-Impfung durch die Krankenkassen von Bedeutung sein. Um diese und weitere mögliche Maßnahmen, wie eine kostenlose, mehrsprachige und aufsuchende Impfberatung und Testung, auszurichten, sollten die Ergebnisse dieser Studie aktualisiert und präzisiert werden.

## Supplementary Information




