# Supplementary material for: Hepatitis B virus infection and vaccine-induced immunity: the role of sociodemographic determinants: Results of the study “German Health Interview and Examination Survey for Adults” (DEGS1, 2008–2011)
Source: Bundesgesundheitsblatt Gesundheitsforschung Gesundheitsschutz. 2021 Dec 27;65(2):159–69. [Article in German] doi: 10.1007/s00103-021-03473-z (PMC8813829; doi:10.1007/s00103-021-03473-z)
Supplement: Supplementary file 1 [file 103_2021_3473_MOESM1_ESM.pdf]

**Bundesgesundheitsblatt – Gesundheitsforschung – Gesundheitsschutz, Ausgabe 2/2022**

Onlinematerial zum Beitrag:

# Hepatitis-B-Virus-Infektionen und impfinduzierte Immunität: die Rolle von soziodemografischen Determinanten. Ergebnisse der „Studie zur Gesundheit Erwachsener in Deutschland“ (DEGS1, 2008-2011)

Annika Brodzinski[1], Angela Neumeyer-Gromen[2], Sandra Dudareva[3], Ruth Zimmermann[3], Ute Latza[4], Viviane Bremer[3], Christina Poethko-Müller[5]

[1] Charité – Universitätsmedizin Berlin (Institut für Hygiene und Umweltmedizin), Berlin, Deutschland
[2] Deutsche Krankenhausgesellschaft e.V. (DKG), Berlin, Deutschland
[3] Robert Koch-Institut (Abteilung für Infektionsepidemiologie), Berlin, Deutschland
[4] Bundesanstalt für Arbeitsschutz und Arbeitsmedizin (BAuA, Fachbereich Arbeit und Gesundheit), Berlin, Deutschland
[5] Robert Koch-Institut (Abteilung für Epidemiologie und Gesundheitsmonitoring), Berlin, Deutschland

**Korrespondenzadresse**
Dr. Christina Poethko-Müller, MSc.
Robert Koch-Institut
Abteilung für Epidemiologie und Gesundheitsmonitoring
FG 25 Körperliche Gesundheit
General-Pape-Straße 62-66
12101 Berlin
Deutschland
poethko-muellerc@rki.de

**Inhalt:**

**Tabelle Z1.** Ausgewählte Berufe des Gesundheitswesens mit erhöhtem Risiko für eine berufliche Exposition gegenüber dem Hepatitis-B-Virus

**Tabelle Z1.** Ausgewählte Berufe des Gesundheitswesens mit erhöhtem Risiko für eine berufliche Exposition gegenüber dem Hepatitis-B-Virus

| Kurz-bezeichnung | Langbezeichnung |
|---|---|
| 8110 | Medizinische Fachangestellte (ohne Spezialisierung) |
| 81102 | Medizinische Fachangestellte (ohne Spezialisierung) – fachlich ausgerichtete Tätigkeiten |
| 81103 | Medizinische Fachangestellte (ohne Spezialisierung) – komplexe Spezialistentätigkeiten |
| 8111 | Zahnmedizinische Fachangestellte |
| 81112 | Zahnmedizinische Fachangestellte – fachlich ausgerichtete Tätigkeiten |
| 81113 | Zahnmedizinische Fachangestellte – komplexe Spezialistentätigkeiten |
| 8112 | Podologen/Podologinnen |
| 81122 | Podologen/Podologinnen – fachlich ausgerichtete Tätigkeiten |
| 8118 | Medizinische Fachangestellte (sonstige spezifische Tätigkeitsangabe) |
| 81182 | Medizinische Fachangestellte (sonstige spezifische Tätigkeitsangabe) – fachlich ausgerichtete Tätigkeiten |
| 81183 | Medizinische Fachangestellte (sonstige spezifische Tätigkeitsangabe) – komplexe Spezialistentätigkeiten |
| 813 | Gesundheits- und Krankenpflege, Rettungsdienst und Geburtshilfe |
| 8130 | Berufe in der Gesundheits- und Krankenpflege (ohne Spezialisierung) |
| 81301 | Berufe in der Gesundheits- und Krankenpflege (ohne Spezialisierung) – Helfer-/Anlerntätigkeiten |
| 81302 | Berufe in der Gesundheits- und Krankenpflege (ohne Spezialisierung) – fachlich ausgerichtete Tätigkeiten |
| 8131 | Berufe in der Fachkrankenpflege |
| 81313 | Berufe in der Fachkrankenpflege – komplexe Spezialistentätigkeiten |
| 8132 | Berufe in der Fachkinderkrankenpflege |
| 81323 | Berufe in der Fachkinderkrankenpflege – komplexe Spezialistentätigkeiten |
| 8133 | Berufe in der operations-/medizintechnischen Assistenz |
| 81332 | Berufe in der operations-/medizintechnischen Assistenz – fachlich ausgerichtete Tätigkeiten |
| 81333 | Berufe in der operations-/medizintechnischen Assistenz – komplexe Spezialistentätigkeiten |
| 8134 | Berufe im Rettungsdienst |
| 81341 | Berufe im Rettungsdienst – Helfer-/Anlerntätigkeiten |
| 81342 | Berufe im Rettungsdienst – fachlich ausgerichtete Tätigkeiten |
| 81343 | Berufe im Rettungsdienst – komplexe Spezialistentätigkeiten |
| 8135 | Berufe in der Geburtshilfe und Entbindungspflege |
| 81352 | Berufe in der Geburtshilfe und Entbindungspflege – fachlich ausgerichtete Tätigkeiten |
| 81353 | Berufe in der Geburtshilfe und Entbindungspflege – komplexe Spezialistentätigkeiten |
| 8138 | Berufe in der Gesundheits- und Krankenpflege (sonstige spezifische Tätigkeitsangabe) |
| 81382 | Berufe in der Gesundheits- und Krankenpflege (sonstige spezifische Tätigkeitsangabe) – fachlich ausgerichtete Tätigkeiten |
| 81383 | Berufe in der Gesundheits- und Krankenpflege (sonstige spezifische Tätigkeitsangabe) – komplexe Spezialistentätigkeiten |
| 8139 | Aufsichts- und Führungskräfte – Gesundheits- und Krankenpflege, Rettungsdienst und Geburtshilfe |

| | |
|---|---|
| 81393 | Aufsichtskräfte – Gesundheits- und Krankenpflege, Rettungsdienst und Geburtshilfe |
| 81394 | Führungskräfte – Gesundheits- und Krankenpflege, Rettungsdienst und Geburtshilfe |
| 814 | Human- und Zahnmedizin |
| 8140 | Ärzte/Ärztinnen (ohne Spezialisierung) |
| 81404 | Ärzte/Ärztinnen (ohne Spezialisierung) - hoch komplexe Tätigkeiten |
| 8141 | Fachärzte/-ärztinnen in der Kinder- und Jugendmedizin |
| 81414 | Fachärzte/-ärztinnen in der Kinder- und Jugendmedizin – hoch komplexe Tätigkeiten |
| 8142 | Fachärzte/-ärztinnen in der Inneren Medizin |
| 81424 | Fachärzte/-ärztinnen in der Inneren Medizin – hoch komplexe Tätigkeiten |
| 8143 | Fachärzte/-ärztinnen in der Chirurgie |
| 81434 | Fachärzte/-ärztinnen in der Chirurgie – hoch komplexe Tätigkeiten |
| 8144 | Fachärzte/-ärztinnen in den Bereichen Hautkrankheiten, Sinnes- und Geschlechtsorgane |
| 81444 | Fachärzte/-ärztinnen in den Bereichen Hautkrankheiten, Sinnes- und Geschlechtsorgane – hoch komplexe Tätigkeiten |
| 8145 | Fachärzte/-ärztinnen in der Anästhesiologie |
| 81454 | Fachärzte/-ärztinnen in der Anästhesiologie – hoch komplexe Tätigkeiten |
| 8146 | Fachärzte/-ärztinnen in der Neurologie, Psychiatrie, Psychotherapie und psychosomatischen Medizin |
| 81464 | Fachärzte/-ärztinnen in der Neurologie, Psychiatrie, Psychotherapie und psychosomatischen Medizin – hoch komplexe Tätigkeiten |
| 8147 | Zahnärzte/-ärztinnen und Kieferorthopäden/-orthopädinnen |
| 81474 | Zahnärzte/-ärztinnen und Kieferorthopäden/-orthopädinnen – hoch komplexe Tätigkeiten |
| 8148 | Ärzte/Ärztinnen (sonstige spezifische Tätigkeitsangabe) |
| 81484 | Ärzte/Ärztinnen (sonstige spezifische Tätigkeitsangabe) – hoch komplexe Tätigkeiten |
| 8149 | Führungskräfte – Human- und Zahnmedizin |
| 81494 | Führungskräfte – Human- und Zahnmedizin |
| 821 | Altenpflege |
| 8210 | Berufe in der Altenpflege (ohne Spezialisierung) |
| 82101 | Berufe in der Altenpflege (ohne Spezialisierung) – Helfer-/Anlerntätigkeiten |
| 82102 | Berufe in der Altenpflege (ohne Spezialisierung) – fachlich ausgerichtete Tätigkeiten |
| 82103 | Berufe in der Altenpflege (ohne Spezialisierung) – komplexe Spezialistentätigkeiten |
| 8218 | Berufe in der Altenpflege (sonstige spezifische Tätigkeitsangabe) |
| 82182 | Berufe in der Altenpflege (sonstige spezifische Tätigkeitsangabe) – fachlich ausgerichtete Tätigkeiten |
| 82183 | Berufe in der Altenpflege (sonstige spezifische Tätigkeitsangabe) – komplexe Spezialistentätigkeiten |
| 8219 | Führungskräfte - Altenpflege |
| 82194 | Führungskräfte - Altenpflege |

Adaptiert nach der “Klassifikation der Berufe 2010” der Bundesagentur für Arbeit [1].

## Literaturverzeichnis

1. Bundesagentur für Arbeit (2013) Klassifikation der Berufe 2010 - Systematisches Verzeichnis. https://statistik.arbeitsagentur.de/Statischer-Content/Grundlagen/Klassifikationen/Klassifikation-der-Berufe/KldB2010/Systematik-Verzeichnisse/Generische-Publikationen/Systematisches-Verzeichnis-Berufsbenennung.xls. Zugegriffen: 28.06.2021
